# Practical Hints

**Published:** 1890-05

**Authors:** George F. Bower


### PRACTICAL HINTS.

By Dr. George F. Bower.[1]

In presenting a paper for your consideration this evening, I thought that it would be as well in this utilitarian age to bring before you for your thoughtful consideration some facts and principles educed from my own observations in the treatment and care of animals, and to invite your attention to the practical side in contrast with the theoretical, and so round out our line of usefulness and conduce to the welfare of the dumb creatures who come under our care, as well as give satisfaction to those who own them and who are often very much attached to them.

It has been my experience in listening to many papers, to notice that the bulk of them have been taken up with the theoretical part of the subject almost wholly, while the practical has been but slightly touched upon and often entirely ignored. A few original home thoughts might with benefit be scattered through once in a while to encourage the talent of the members for development of the useful side and let the German with his "jaw-breaking unpronounceables" have a rest for a while. Perhaps we may be able to give him a few points of our own cultivation to help him out. I would not have you think from these remarks that I do not think highly of the theory, or that I am here to belittle such efforts, for that concerns the cause, and getting the cause we can readily find the means to help, but how often has it been noticed that the practice though in the dark has thrown a flood of light on the way to the cause, and both have worked in harmony together to establish a truth—both are necessary to round out a full and perfect picture that shall reflect the best.

Have we not seen that many of the remedies that have been used with a lavish hand have been by experience shown to be useless as well as harmful and must be relegated as rubbish to the darker ages. The blind handings-down from the father blacksmith to his son of his stock of remedies has given place to an enlightened course of genuine scientific procedure. Now, for

[1] Delivered before the Long Island Veterinary Society at its February meeting, 1890.

example, quinine has been thought to do good in inflammatory troubles from the idea that it will lower the temperature. This idea was orignally taken probably from the medical authorities who gave out that it did so in human subjects. That idea is in good part dispelled by a riper experience, and my observation tends to the opinion that in reference to high temperature in animals it may safely be abandoned. I have tested this in pneumonia, side by side in the same stable and under exactly similar conditions. In the giving of quinine in one set and its entire absence in others, I have noticed the same rise and fall of the thermometer in one as in the others, and in large doses a dulness of eye and stupidity of action in those taking quinine that was not noticed in the others. Those not taking quinine convalesced quicker than those who did, so the conclusion is forced upon me that as far as rise and fall of temperature is concerned, quinine is *nil*, and that the slow convalescence spoken of above. was due, as I think, to the tax on the nervous system in eliminating the remedy.

Now, you will no doubt ask what I propose to use in place of quinine. I have found that acetanilide and digitalis in combination, and at times aconite, will give me excellent results. They can be given in small doses, are easy of administration and in every way are more satisfactory. Now, as to the usefulness of quinine when not given directly for its action on the temperature, I have found this remedy useful as a tonic, an appetizer and also as an anti-ferment.

While on the subject of pneumonia, I would like to say a few words on counter-irritation. The usual custom, as you know, is to use mustard to blister. Now, I have found that this action tends to produce a want of freedom in respiration, perhaps by the soreness induced, and the animal seems not to enjoy its breathing as well as without it. I use ammonia to irritate, not to blister, and then hot applications and some sort of waterproof covering and with excellent results. Internally it has been my habit to give fluid forms of medicine, for the reason that there seems to be a torpidity of the mucous membranes, and fluids were more readily absorbable than solids. In the class of cases above mentioned, aromatic spirits of ammonia and whiskey have been used by me with good results, and this plan of treatment has seemed in full accordance with the observation of many others, who have found that the absorbent surfaces in disease were illy adapted to take up very much of anything, and, especially if it first had to be dissolved.

I would like to call the attention of the members to an interesting case that has lately come under my care, thinking that it may be of as much interest to the society as it was to me. I was called to see a horse whose main symptom consisted of rejection of its food. No other functional or organic disturbance existed, and my mind led me to the idea that it was of an obstructive nature, the result of greediness in eating. The remedy seemed to me to be the opening of the gullet and the pushing of the offending material onward. This was refused by the owner. On consultation with a fellow practitioner, who concurred in the diagnosis he recommended blistering the inferior portion of the neck and bandaging the animal's legs, and applying additional clothing and withholding all food and drink. I could not see the advisability of this course, but ordered it to be done from proper courtesy. The animal died. Post-mortem showed firm obstruction near the stomach of mixed hay and oats masticated. Independent of an operation, my plan would have been to have passed bougies, and failing in that to have passed a faradic current through the parts, with the endeavor to promote contraction of muscles and so force the obstruction onward.

There is another class of cases that I would like to call attention to, viz.: The trouble in the bladder in cases of paralysis. I have had lately two cases of the kind which have been exceedingly interesting to me, symptoms as you well know of frequent micturition, passage of offensive mucus, the pathological conditions being an inflamed, thickened membrane, coated with mucus and pus, and at times ulcerations.

One case now under treatment of twelve months' duration was not benefited in any respect, until, conceiving the idea that dilatation and washing would benefit, I put the animal under such treatment. By dilatation, I mean forced stretching of the mucous membrane. This had a very happy effect, and as I despaired of the animal's life before, I now think he will get well, and, as a reason therefor, that the stretching of the rugæ allowed the antiseptic wash used to cleanse the parts, removing stagnate and decomposed mucus, and thus allaying the inflammation by removing the irritating cause. Then the vis medicatrix naturæ had full play and the animal improved, passed much less water and picked up in health and strength. Not to tire your patience farther, and thanking you for the attention bestowed, I will now leave the subject and present the paper to you for criticism, for after all it is by persistent examinations and discussions of the pros and cons that truth can be arrived at and axioms drawn therefrom.

